# Oxidative Folding of a Two‐Chain Protein Having Three Interchain Disulfide Bonds. Synthesis of Bromelain Inhibitor VI Through Native Chain Assembly

**DOI:** 10.1002/chem.202500486

**Published:** 2025-05-20

**Authors:** Michio Iwaoka, Sawa Akaboshi

**Affiliations:** ^1^ Department of Chemistry School of Science Tokai University Kitakaname Hiratsuka‐shi Kanagawa 259–1292 Japan; ^2^ Institute of Advanced Biosciences Tokai University Kitakaname Hiratsuka‐shi Kanagawa 259–1292 Japan

**Keywords:** 7‐amino‐4‐methylcoumarin (AMC), dmb protection, multichain protein, oxidative protein folding, solid‐phase peptide synthesis

## Abstract

The recently renovated two‐chain folding method, in which two native peptide chains without any sidechain protections and interchain scaffolds are just mixed in a buffer solution under optimized conditions, called native chain assembly (NCA), enabled efficient chemical synthesis of α‐helix‐rich insulin and its analogs, which are stabilized by two interchain disulfide (SS) bridges. Herein, this simple folding method has been successfully applied to the folding of a different‐type two‐chain protein, that is, bromelain inhibitor VI (BI‐VI), which has abundant β‐sheet structures and is stabilized by three interchain SS bridges. When the chemically synthesized native heavy (H)‐ and light (L)‐chains of BI‐VI were mixed at 4 °C in a pH of 10.0 buffer solution containing 2 mM GSH and 0.4 mM GSSG, native BI‐VI was obtained surprisingly in a high isolated yield (53%) after 2 weeks. The obtained BI‐VI showed complete inhibitory activity against bromelain, whereas each component chain exhibited essentially non‐activity. The rate‐limiting step of the two‐chain folding was assumed to be the chain coupling between three‐SS intermediates of the H‐chain (3SS^H^) and one‐SS intermediates of the L‐chain (1SS^L^). This achievement opens a door to the chemical synthesis of unprecedent multichain proteins with more complicated SS‐bond topologies.

## Graphical abstract

Bromelain inhibitor VI, a two‐chain protein stabilized by three interchain SS bonds and two intrachain SS bonds, was synthesized in an incredibly high isolated yield (53%) just by mixing the two native chains in a buffer solution. This success made a record of oxidative protein folding for the most complicated and difficult one.

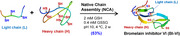

## 1 Introduction

Protein folding is an important in vivo and in vitro process, in which a nascent or unfolded polypeptide chain gains its biological functions as a protein. It is well accepted by the researchers that this process proceeds spontaneously toward the specific folded structure, called “a native state”, depending only on the amino acid sequence.^[^ ^1^ ^]^ Although the prediction of a protein structure from the amino acid sequence, hence the artificial design of functional proteins, has recently become a practical research tool,^[^ ^2^, ^3^, ^4^ ^]^ it is still challenging in laboratories to fold a protein properly from the unfolded polypeptide chain, in particular for those having an intricate disulfide (SS)‐bond topology.^[^ ^5^, ^6^, ^7^, ^8^, ^9^ ^]^

**Figure 1:**
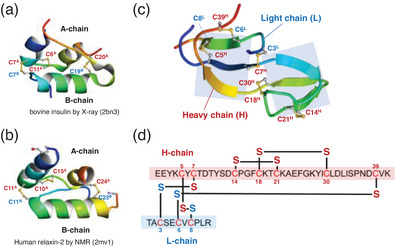
Representative two‐chain proteins. Molecular structures of (a) bovine insulin determined by X‐ray analysis (PDBID: 2BN3), (b) human relaxin‐2 determined by NMR (PDBID: 2MV1), and (c) bromelain inhibitor VI (BI‐VI) determined by NMR (PDBID: 2BI6). (d) The primary amino acid sequence and the SS‐bond topology of BI‐VI.

In the oxidative folding of SS‐containing proteins, the native state is gradually generated from the fully reduced state through the oxidation of cysteinyl thiols (SHs), i.e., SS formation, and the reshuffling of the SS bonds, that is, SS rearrangement. In the chemical synthesis of such proteins, the control of these processes is a key factor to increase the yield of the native state.^[^ ^10^, ^11^, ^12^, ^13^ ^]^ Indeed, the folding yields of single‐chain proteins having multiple SS bonds can be increased in more than 90% by optimizing the folding conditions.^[^ ^14^, ^15^ ^]^ On the other hand, for the case of two‐chain proteins, such as insulins, the folding yields were very low due to the preference of intramolecular SS formation to intermolecular SS formation. For example, the yields of native insulins were less than 5% by just mixing the A‐ and B‐chains in a buffer solution.^[^ ^16^, ^17^, ^18^, ^19^ ^]^ To overcome this problem in the folding of two‐chain proteins, the SH groups of each peptide chain should be protected orthogonally with different groups, and the selective deprotection and subsequent intermolecular SS formation should be applied.^[^ ^20^, ^21^, ^22^, ^23^ ^]^ Alternatively, the two peptide chains should be chemically connected with a linker, and after the oxidative folding, the scaffold is cut off to afford the native protein.^[^ ^24^, ^25^, ^26^ ^]^ Recently, it has been reported that introduction of selenocysteine residues, instead of the cysteine residues, is a useful strategy not only to increase the folding efficiency for insulins but also to confer new biological functions to them.^[^ ^27^, ^28^, ^29^, ^30^ ^]^ Moreover, our recent reinvestigation on the mixing conditions for the two native chain folding of insulins revealed that this simple classical method can be renovated so that the native human insulin is isolated in a yield of up to 49% under optimized conditions.^[^ ^19^ ^]^ This technique, called “native chain assembly (NCA)”, relies on applications of low temperature, optimal pH, adequate SS‐forming reagents, and additives. By applying NCA, highly efficient total chemical synthesis of human relaxin‐2 (49%),^[^ ^19^ ^]^ selenoinsulin (72%),^[^ ^29^ ^]^ and selenorelaxin (73%)^[^ ^31^ ^]^ were also achieved. However, insulin and relaxin belong to the same superfamily and have similar α‐helix‐rich structures (Figure 1a,b). It was not clear whether NCA is applicable to the other types of two‐chain proteins with completely different folded structures.

Bromelain inhibitors (BIs) are a family of proteins isolated from pineapple stems.^[^ ^32^ ^]^ The biological function is to inhibit cysteine protease bromelain, which is present in pineapple fruits, stems, and leaves. Since bromelain has anti‐inflammatory properties and has long been used as a medicine to treat sick conditions, such as arthritis, sinusitis, and inflamed bronchitis,^[^ ^33^, ^34^ ^]^ BIs would be important pharmaceuticals to control the drug efficacy of bromelain. Bromelain Inhibitor VI (BI‐VI) (Figure 1 c,d) is a representative member of the BI family. This SS‐rich small protein with a molecular weight of approximately 6000 is abundant of β‐sheet structures. The protein consists of two peptide chains: a heavy chain (H‐chain) of 41 amino acid residues and a light chain (L‐chain) of 11 amino acid residues.^[^ ^35^, ^36^, ^37^ ^]^ There are seven cysteine residues in the H‐chain and three in the L‐chain. The molecular structure is stabilized by five SS bonds (C5^H^‐C8^L^, C7^H^‐C3^L^, C14^H^‐C21^H^, C18^H^‐C30^H^, and C39^H^‐C6^L^), three of which are formed between the two peptide chains. In vitro folding of a multichain protein having more than three interchain SS bonds has never been succeeded due to its complicated SS‐bond topology. Thus, BI‐VI is an interesting and challenging target in chemical synthesis of proteins. In this study, oxidative folding of BI‐VI was studied by applying NCA to demonstrate the versatile usability of NCA for multichain proteins having complicated interchain SS linkage patterns.

## 2 Results and Discussion

**Figure 2:**
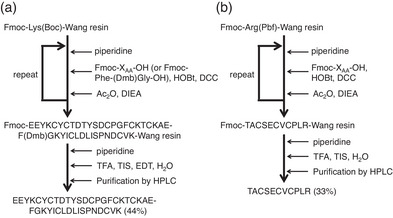
SPPS protocols for the H‐ and L‐chains of BI‐VI. (a) Synthesis of H‐chain applying (Dmb)Gly. (b) Synthesis of L‐chain. HOBt, 1‐hydroxybenzotriazole; DCC, *N*,*N*'‐dicyclohexylcarbodiimide; Ac_2_O, acetic anhydride; DIEA, *N*,*N*‐diisopropylethylamine; TFA, trifluoroacetic acid; TIS, triisopropylsilane; EDT, 1,2‐ethanedithiol.

The H‐ and L‐chains were synthesized by the solid‐phase peptide synthesis (SPPS) method applying Fmoc protection (Figure 2). In our initial attempt, however, the H‐chain was not obtained by the normal SPPS protocol. Instead, a 20‐mer peptide (Ac‐KAEFGKYIC^30^LDLISPNDC^39^VK) was obtained after the cleavage from the resin (Figure S1). Since BI‐VI is a β‐rich protein, it was deduced that the elongation of the peptide chain was hindered by formation of a local β‐sheet structure on the resin, which would sterically bury the reactive site at the end of the growing peptide chain. Therefore, we introduced *N*‐(2,4‐dimethoxybenzyl)glycine ((Dmb)Gly), which would prohibit the formation of a hydrogen bond hence β sheet, instead of Gly26. To do this, dipeptide Fmoc‐Phe‐(Dmb)Gly‐OH^[^ ^38^, ^39^ ^]^ was synthesized and manually coupled to the Lys27 of the growing peptide chain on the resin. Indeed, the introduction of the Dmb‐protected glycine residue overcame the problem in the SPPS, and the H‐chain was successfully isolated in a yield of 44% after the cleavage of the peptide from the resin using the trifluoroacetic acid (TFA) cocktail, followed by purification (Figure S2). For the synthesis of the L‐chain, the aimed 11‐mer peptide was obtained in a yield of 33% by following the normal SPPS protocol (Figure S3). The purity and identities of these peptide chains were confirmed by amino acid analysis as well as HPLC and MS analyses.

**Figure 3:**
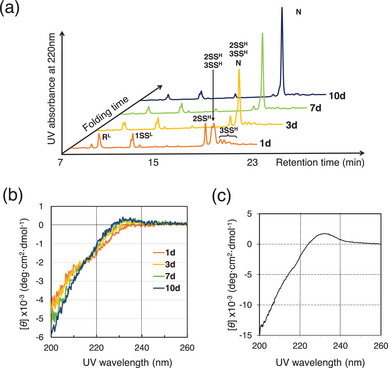
A time‐course of the oxidative folding of BI‐VI via native chain assembly (NCA). (a) A series of HPLC chromatograms. Folding conditions were at pH of 10.0 and 4 °C in a 25 mM sodium bicarbonate buffer solution in the presence of 2 mM GSH and 0.4 mM GSSG as a redox buffer. The reaction was quenched with AEMTS. HPLC analysis conditions: column, a TSKgel ODS‐100 V φ 4.6 × 150 mm RP column (Tosoh Corporation, Japan); detection, at 220 nm; solvent gradient, CH_3_CN 10 to 40% in 25 minutes in the presence 0.1% TFA; flow rate, 1.0 mL min^−1^. R^H^, 2SS^H^, and 3SS^H^ represent the reduce, two‐disulfide bonded, and three‐disulfide bonded intermediates of the H‐chain. R^L^ and 1SS^L^ represent the reduce and one‐disulfide bonded intermediate of the L‐chain. N represents the native state. (b) CD spectral changes measured at pH of 3.9 and 37 °C in the presence of 0.1 M NaCl. Folding conditions were the same as (a). (c) CD Spectrum of BI‐VI isolated after purification. Details for the CD spectrum measurement conditions are to see experimental section.

Having obtained the native peptide chains, we subsequently attempted to fold BI‐VI by applying the modified NCA conditions. Thus, the non‐protected (or native) H‐ and L‐chains were mixed under several NCA conditions (Tables 1 and S1). After certain periods of time, the reaction mixture was added with 2‐aminoethyl methanethiosulfonate (AEMTS) to quench the folding reaction. AEMTS blocks free SH groups to SSCH_2_CH_2_NH_3_^+^,^[^ ^40^ ^]^ allowing good peak separation on an HPLC chart and easy structural characterization by mass spectrometry for the folding intermediates. The representative HPLC chromatograms that were obtained during the folding of BI‐VI at pH of 10.0 and 4 °C in the presence of 2 mM GSH and 0.4 mM GSSG as a redox buffer (under the same conditions as Table 1, entry 3) are shown in Figure 3, along with the corresponding CD spectral changes. Under these conditions, the native species (N) was not generated in the first 2 days, during which slow oxidations of the reduced H‐chain (R^H^) and L‐chain (R^L^) proceeded to accumulate two‐ and three‐disulfide intermediates of the H‐chain (2SS^H^ and 3SS^H^) and one‐disulfide intermediates of the L‐chain (1SS^L^) (see also Figures S4 and S6). It should be noted that these partially oxidized intermediates were obtained as an ensemble of various SS‐bonded species.

**Table 1:** Summary of oxidative two‐chain folding of BI‐VI via native chain assembly (NCA).^a)^

Entry	H‐chain (µM)	L‐chain (µM)	pH	Temp (°C)	Additives	Reaction Time	Isolated Yield (%)	HPLC Yield (%)
1	200	200	10	‒10	0.4 M urea, 4 mM GSH, 0.8 mM GSSG, 5 mM DTT, 10% EG	7 w	28	47
2	200	200	10	‒10	2 mM GSH, 0.4 mM GSSG, 10% EG	4 w	32	58
3	200	200	10	4	2 mM GSH, 0.4 mM GSSG	2 w	53	83
4	200	200	10	4	1.0 mM DTT	4 w	36	72

^a)^ GSH, glutathiones; GSSG, glutathione disulfide; DTT, dithiothreitol; EG, ethylene glycol.

Following these pre‐folding events, N was slowly generated after 3 days probably through the coupling between 3SS^H^ and 1SS^L^. The CD spectrum also started to change gradually in consonance with the generation of N. A small positive cotton effect was observed at around 230 nm, which was also characteristic to native BI‐VI.^[^ ^41^ ^]^ After 10 days, the peak corresponding to N became clear on the HPLC chart. This peak was collected and analyzed by MALDI‐TOF‐MS and CD as well as amino acid analysis. The results unambiguously confirmed that N is native BI‐VI (Figures 3c and S5).

The representative results of the oxidative folding of BI‐VI under various NCA conditions are summarized in Table 1. When the folding was carried out under the standard conditions of NCA for insulin, i.e., at pH of 10.0 and ‒10 °C in the presence of urea (0.4 M), GSH (4 mM), GSSG (0.8 mM), and DTT (5 mM) (entry 1), the reaction proceeded very slowly, and N was isolated in a yield of 28% after 7 weeks. Since each H‐ and L‐chain did not aggregate in the absence of urea, the folding was also carried out without using urea (entry 2). The yield of N increased slightly up to 32% after 4 weeks. To increase the reaction velocity, we subsequently raised the temperature to 4 °C in the absence of DTT (entry 3). This resulted in a significant increase in the reaction yield up to 53% after 2 weeks. However, when DTT was employed instead of GSH/GSSG as a redox buffer (entry 4), the yield of N decreased. Results of other entries for the oxidative folding of BI‐VI are provided in Table S1, along with series of HPLC charts obtained with different folding times (Figure S6). Accordingly, the best NCA conditions for BI‐VI was determined to be at pH 10.0 and 4 °C in the presence of 2 mM GSH and 0.4 mM GSSG (entry 3). The yield of 53% was the best yield in the oxidative folding of two‐chain wild‐type proteins via NCA (*cf*. 49 and 47% for human insulin and human relaxin‐2, respectively).^[^ ^19^ ^]^ It should be noted that the yield of BI‐VI can be improved in light of the yield of 83% that was estimated from the HPLC chart of the folding mixture.

**Figure 4:**
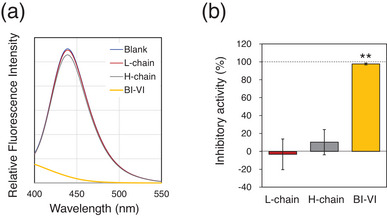
Inhibitory assays of synthesized BI‐VI against bromelain using Boc‐Leu‐Arg‐Arg‐AMC (MCA) as a substrate. (a) Fluorescence spectra with an excitation wavelength of 370 nm. Assay conditions were 5 µM test sample (BI‐VI, H‐chain, or L‐chain), 20 nM bromelain, 50 µM MCA, and 0.5 mM 2‐mercaptoethanol. The spectra were measured 10 min after the initiation of the reaction. (b) Inhibitory activities of the test samples against bromelain estimated by the decrease of fluorescent at 460 nm. Error bars are indicated as Mean ± SD (*n* = 3). See the experimental section for details of the assay conditions. One‐way ANOVA and the post‐hoc Tukey test were performed to evaluate the statistical difference between the groups (** *p* < 0.01).

To characterize the obtained BI‐VI in terms of the biological function, the inhibitory activity against bromelain was assayed using the 7‐amino‐4‐methylcoumarin (AMC)‐labeled tripeptide, Boc‐Leu‐Arg‐Arg‐AMC (MCA), as a substrate of the enzyme (Figure 4). AMC shows strong fluorescence at 440 nm. Therefore, when MCA is hydrolyzed by bromelain, fluorescence can be observed due to generation of AMC.^[^ ^32^ ^]^ The results of this assay clearly indicated that the synthesized BI‐VI almost completely inhibits bromelain (98 ±1% inhibition), which was same as that reported previously,^[^ ^32^ ^]^ whereas the individual H‐ and L‐chains do not inhibit the enzyme (10 ±14 and –3 ±17% inhibition, respectively) (see also Figure S7). Thus, it is obvious that the native folded structure of BI‐VI, which is stabilized by three interchain SS bridges, is essential for the inhibition against bromelain. On the other hand, it was previously reported that the oxidized H‐ and L‐chains of BI‐III showed significant inhibitory activity (ca. 20% and >70%, respectively),^[^ ^32^ ^]^ suggesting that the individual H‐ and L‐chains possess native‐like structures to some extent in the oxidized state.

**Figure 5:**
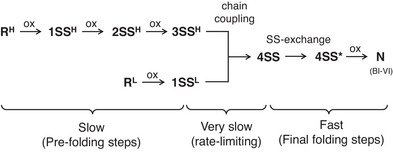
A plausible major oxidative folding pathway of BI‐VI.

As for the oxidative two‐chain folding pathway of BI‐VI, the following scenario (Figure 5) is assumable based on the changes of the HPLC chromatograms and CD spectra observed during the NCA (Figures 3 and S6). The folding pathway consists of three phases. The first phase corresponds to slow prefolding steps, during which the H‐ and L‐chains are slowly oxidized intramolecularly to generate 3SS^H^ and 1SS^L^ intermediates, respectively, which have native‐like structure to some extent.^[^ ^32^ ^]^ Similar prefolding events were observed in the oxidative folding of two‐chain insulin and relaxin.^[^ ^19^, ^42^ ^]^ The second phase would be a very slow or rate‐limiting chain coupling step between 3SS^H^ and 1SS^L^ forming an ensemble of the cross‐linked 4SS intermediates having four SS bonds. The last phase would be the conformational folding step, in which the 4SS intermediates rearrange their SS bonds to obtain four native SS bonds and then the generated 4SS* species are oxidized to N by formation of the last native SS bond. Since no folding intermediate, which possess a molecular mass corresponding to 4SS and 4SS*, was observed on the HPLC chromatograms during the folding of BI‐VI, the last oxidation step to N rapidly proceeds under the NCA conditions. This folding pathway relies on the thermodynamic stability of the native folded structure of BI‐VI, as previously observed in the oxidative folding of various SS‐containing proteins.

## 3 Conclusions

We have succeeded in the total chemical synthesis of BI‐VI in the isolated yield of up to 53%, from the ingredient H‐ and L‐chains, by applying the NCA method. This is the first achievement in the oxidative folding of a two‐chain protein having three interchain SS linkages. Thus, it is proved that the NCA method is useful not only for α‐rich two‐chain proteins like insulins but also for the other‐type two‐chain protein, that is, BI‐VI, which is rich in β‐sheet structure. Notably, the occurrence of deamidation of the peptide chains was not observed during NCA in spite of the long reaction time. This would be due to the applied low temperatures and is an advantage of NCA as an oxidative folding methodology. It was also proposed that in the oxidative folding of BI‐VI the rate‐limiting step is the chain coupling between the 3SS^H^ and 1SS^L^ intermediates under the NCA conditions. Isolation and structural characterization of the key folding intermediates, i.e., 4SS and 4SS*, will elucidate the two‐chain folding pathway of BI‐VI more clearly. The achievements of this study will open the door to the total chemical synthesis of multi‐chain proteins with more complicated SS‐bond topologies.

## 4 Experimental Section

### General

2‐Aminoethyl methanethiosulfonate (AEMTS) was synthesized according to the literature method.^[^ ^43^ ^]^ All other general reagents were commercially available and used without further purification. ^1^H NMR spectra were measured at 500 MHz on a Bruker AV‐500 spectrometer at 298 K in CDCl_3_ using the solvent signal as an internal standard of the chemical shifts. H‐ and L‐chains of BI‐VI were synthesized by Fmoc solid‐phase peptide synthesis (SPPS) using microwave peptide synthesizer Initiator+ (Biotage, Japan) or manually. The two‐chain folding intermediates as well as N (native BI‐VI) were isolated by reverse‐phase (RP)‐HPLC after quenching the folding with AEMTS, and the structures were characterized by MALDI‐TOF‐MS using α‐cyano‐4‐chlorocinnamic acid as a matrix or ESI‐TOF‐MS using water as a solvent, after lyophilizing the collected solutions. The amino acid components as well as the yields of the isolated H‐ and L‐chains and N were determined by amino acid analysis (AAA) by hydrolyzing the sample with 6 M HCl at 200 °C for 2 h.

### Synthesis of Fmoc‐Phe‐(Dmb)Gly‐OH

According to the literature procedure,^[^ ^38^ ^]^ Fmoc‐Phe‐(Dmb)Gly‐OH was synthesized as follows. The synthesis consists of two steps. In the first step, *N*‐(9‐fluorenylmethoxycarbonyl)‐*N*‐(2,4‐dimethoxybenzyl)glycine (Fmoc‐(Dmb)Gly‐OH, 100.5 mg, 0.22 mmol) was reacted with diethylamine (0.45 mL) in dichloromethane (DCM) (4.5 mL) for 2.5 hours at 40 °C to afford H‐(Dmb)Gly‐OH (61.7 mg, quant) after purification by silica gel column chromatography. ^1^H NMR (500 MHz, in CDCl_3_) δ 7.19 (m, 1H), 6.36 (m, 1H), 6.35 (s, 1H), 4.01 (s, 2H), 3.72 (s, 3H), 2.70 (s, 3H), 3.28 (s, 2H). In the second step, to the solution of H‐(Dmb)Gly‐OH (3.0 mg) in DCM (5 mL) was added *N*,*O*‐bis(trimethylsilyl)acetamide (8.3 µL, 2.5 eq). After stirring for 30 minutes at room temperature, the resulting mixture was added with a solution of *N*‐(9‐fluorenylmethoxycarbonyl)‐L‐phenylalanine pentafluorophenyl ester (Fmoc‐Phe‐OPfp, 7.4 mg, 1 eq) in DCM (0.1 mL) and *N*,*N*‐diisopropylethylamine (DIEA) (0.5 µL, 0.2 eq). The mixture was stirred for 17 hours at room temperature. After removal of the solvent under a reduced pressure, the crude products were purified by silica gel column chromatography to obtain Fmoc‐Phe‐(Dmb)Gly‐OH (6.3 mg, 70%). ^1^H NMR (500 MHz, in CDCl_3_) δ 7.8‒6.7 (m, 14H), 6.40 (m, 2H), 6.03–5.90 (m, 1H), 5.31 (m, 1H), 4.8–4.0 (m, 6H), 3.82‒3.70 (m, 7H), 3.17–2.94 (m, 2H). The observed spectrum was consistent with that reported in a recent literature.^[^ ^44^ ^]^ The synthesis was repeated several times in a larger scale.

### SPPS of BI‐VI H‐Chain

Fmoc‐Lys(Boc)‐Wang resin (0.56 mmol/g, 180 mg, 0.10 mmol) was swelled with DCM (3 mL) for 3 hours. After washing with *N*‐methyl‐2‐pyrrolidone (NMP) for 1 minute (3 mL×4), the peptide chain was grown on the resin by the Fmoc‐SPPS method till Lys27 using a microwave peptide synthesizer as follows. First, the Fmoc group of the resin was removed by treating with 20% piperidine/NMP (4.5 mL) for 5 and 15 minutes. After washing with NMP for 1 minute (3 mL×4), the resin was coupled at 75 °C for 5 min under microwave irradiation with an Fmoc‐protected amino acid (0.50 mmol), which had been activated with 0.5 M *N*,*N*’‐dicyclohexylcarbodiimide (DCC)/NMP (1 mL) and 0.5 M 1‐hydroxybenzotriazole (HOBt)/NMP (1 mL). In the coupling with Cys, the resin was reacted with the activated Fmoc‐Cys(Trt)‐OH at room temperature for 1 hour without microwave irradiation. The resin was washed with 50% MeOH/DCM for 45 seconds (3 mL×3), reacted with 5 M Ac_2_O/NMP and 2 M DIEA/NMP for 10 minutes, and then washed with NMP for 1 minute (3 mL×4). This coupling procedure was repeated for Fmoc‐Val‐OH, Fmoc‐Cys(Trt)‐OH, Fmoc‐Asp(OtBu)‐OH, Fmoc‐Asn(Trt)‐OH, Fmoc‐Pro‐OH, Fmoc‐Ser(tBu)‐OH, Fmoc‐Ile‐OH, Fmoc‐Leu‐OH, Fmoc‐Asp(OtBu)‐OH, Fmoc‐Leu‐OH, Fmoc‐Cys(Trt)‐OH, Fmoc‐Ile‐OH, Fmoc‐Try(tBu)‐OH, and Fmoc‐Lys(Boc)‐OH. The obtained resin was washed with NMP for 1 min (3 mL×4), treated with 20% piperidine/NMP (4.5 mL) for 5 and 15 minutes, washed with DCM for 1 minutes (3 mL×6), and dried under vacuum for 2 hours.

The obtained H‐Lys(Boc)‐Tyr(tBu)‐Ile‐Cys(Trt)‐Leu‐Asp(OtBu)‐Leu‐Ile‐Ser(tBu)‐Pro‐Asn(Trt)‐Asp(OtBu)‐Cys(Trt)‐Val‐Lys(Boc)‐Wang resin was manually coupled at 50 °C for 1.5 hours with Fmoc‐Phe‐(Dmb)Gly‐OH (260 mg, 0.437 mmol), which had been activated with DCC (125 mg, 0.6 mmol) and HOBt (82 mg, 0.6 mmol) in NMP (3 mL) at room temperature for 30 minutes. The resin was then washed with NMP for 1 minute (3 mL×4), treated with 20% piperidine/NMP (4.5 mL) for 5 and 15 minutes, washed with DCM for 1 minute (3 mL×6), and dried under vacuum for 2 hours. To confirm the completion of the coupling reaction, a small portion of the resin was treated with a TFA cocktail (TFA:TIS:H_2_O = 36:1:1) to cleave the peptide from the resin. The peptide was analyzed by RP‐HPLC and MALDI‐TOF‐MS, which revealed that H‐Phe‐(Dmb)Gly‐Lys(Boc)‐Tyr(tBu)‐Ile‐Cys(Trt)‐Leu‐Asp(OtBu)‐Leu‐Ile‐Ser(tBu)‐Pro‐Asn(Trt)‐Asp(OtBu)‐Cys(Trt)‐Val‐Lys(Boc)‐Wang resin was obtained with a reasonable purity.

Subsequently, one third of the resin was taken and coupled with a series of Fmoc‐protected amino acids using the peptide synthesizer with the same procedure as mentioned above. Thus, Fmoc‐Glu(OtBu)‐OH, Fmoc‐Ala‐OH, Fmoc‐Lys(Boc)‐OH, Fmoc‐Cys(Trt)‐OH, Fmoc‐Thr(tBu)‐OH, Fmoc‐Lys(Boc)‐OH, Fmoc‐Cys(Trt)‐OH, Fmoc‐Phe‐OH, Fmoc‐Gly‐OH, Fmoc‐Pro‐OH, Fmoc‐Cys(Trt)‐OH, Fmoc‐Asp(OtBu)‐OH, Fmoc‐Ser(tBu)‐OH, Fmoc‐Try(tBu)‐OH, Fmoc‐Thr(tBu)‐OH, Fmoc‐Asp(OtBu)‐OH, Fmoc‐Thr(tBu)‐OH, Fmoc‐Cys(Trt)‐OH, Fmoc‐Try(tBu)‐OH, Fmoc‐Cys(Trt)‐OH, Fmoc‐Lys(Boc)‐OH, Fmoc‐Try(tBu)‐OH, Fmoc‐Glu(OtBu)‐OH, and Fmoc‐Glu(OtBu)‐OH were sequentially reacted. The obtained resin was washed with NMP for 1 min (3 mL×4), treated with 20% piperidine/NMP (4.5 mL) for 5 and 15 min, washed with DCM for 1 minute (3 mL×6), and dried under vacuum for 2 hours. H‐Glu(OtBu)‐Glu(OtBu)‐Try(tBu)‐Lys(Boc)‐Cys(Trt)‐Try(tBu)‐Cys(Trt)‐Thr(tBu)‐Asp(OtBu)‐Thr(tBu)‐Try(tBu)‐Ser(tBu)‐Asp(OtBu)‐Cys(Trt)‐Pro‐Gly‐Phe‐Cys(Trt)‐Lys(Boc)‐Thr(tBu)‐Cys(Trt)‐Lys(Boc)‐Ala‐Glu(OtBu)‐Phe‐(Dmb)Gly‐Lys(Boc)‐Tyr(tBu)‐Ile‐Cys(Trt)‐Leu‐Asp(OtBu)‐Leu‐Ile‐Ser(tBu)‐Pro‐Asn(Trt)‐Asp(OtBu)‐Cys(Trt)‐Val‐Lys(Boc)‐Wang resin (290.9 mg) was obtained.

A portion of the resin (29.5 mg, 3.38 µmol) was treated with a TFA cocktail (TFA:TIS:H_2_O:1,2‐ethanedithiol (EDT) = 37:1:1:1, 600 µL) at room temperature for 2 hours. The cleaved peptide was precipitated with cold diethyl ether (14 mL). After washing three times with diethyl ether, the precipitate was dried under vacuum. The crude peptide was purified by RP‐HPLC to afford H‐Glu‐Glu‐Try‐Lys‐Cys‐Try‐Cys‐Thr‐Asp‐Thr‐Try‐Ser‐Asp‐Cys‐Pro‐Gly‐Phe‐Cys‐Lys‐Thr‐Cys‐Lys‐Ala‐Glu‐Phe‐Gly‐Lys‐Tyr‐Ile‐Cys‐Leu‐Asp‐Leu‐Ile‐Ser‐Pro‐Asn‐Asp‐Cys‐Val‐Lys‐OH (1.49 µmol, 44% as an overall yield from the resin loaded). ESI‐TOF mass, found: *m*/*z* 1175.35, calcd: 1175.26 for [M+4H]^4+^. Amino acid analysis: Asp_5.15_ Thr_2.53_ Ser_1.86_ Glu_3.15_ Pro_1.59_ Gly_1.97_ Ala_1_ Val_1.00_ Ile_1.92_ Leu_2.13_ Tyr_3.62_ Phe_1.99_ Lys_4.19_.

### SPPS of BI‐VI L‐Chain

Fmoc‐Arg(Pbf)‐Wang resin (0.57 mmol/g, 180 mg, 0.10 mmol) was swelled with DCM (3 mL) for 3 hours. After washing with NMP for 1 minute (3 mL×4), the peptide chain was grown on the resin by the Fmoc‐SPPS method using a microwave peptide synthesizer, following the same protocol as applied in the synthesis of the H‐chain. After the sequential reactions with Fmoc‐Leu‐OH, Fmoc‐Pro‐OH, Fmoc‐Cys(Trt)‐OH, Fmoc‐Val‐OH, Fmoc‐Cys(Trt)‐OH, Fmoc‐Glu(OtBu)‐OH, Fmoc‐Ser(tBu)‐OH, Fmoc‐Cys(Trt)‐OH, Fmoc‐Ala‐OH, and Fmoc‐Thr(tBu)‐OH, the obtained resin was washed with NMP for 1 minute (3 mL×4), treated with 20% piperidine/NMP (4.5 mL) for 5 and 15 minutes, washed with DCM for 1 minute (3 mL×6), and dried under vacuum for 2 hours. H‐Thr(tBu)‐Ala‐Cys(Trt)‐Ser(tBu)‐Glu(OtBu)‐Cys(Trt)‐Val‐Cys(Trt)‐Pro‐Leu‐Arg(Pbf)‐Wang resin (365.6 mg) was obtained.

A portion of the resin (20.0 mg, 5.47 µmol) was treated with a TFA cocktail (TFA:TIS:H_2_O = 48:1:1, 1 mL) at room temperature for 2 hours. The cleaved peptide was precipitated with cold diethyl ether (14 mL). After washing three times with diethyl ether, the precipitate was dried under vacuum. The crude peptide was purified by RP‐HPLC to afford H‐Thr‐Ala‐Cys‐Ser‐Glu‐Cys‐Val‐Cys‐Pro‐Leu‐Arg‐OH (1.81 µmol, 33% as an overall yield from the resin loaded). MALDI‐TOF mass, found: *m*/*z* 1181.76, calcd: 1181.51 for [M+H]^+^. Amino acid analysis: Thr_0.93_ Ser_0.87_ Glu_1.10_ Pro_0.97_ Ala_1_ Val_0.94_ Leu_0.92_ Arg_1.01_.

### Oxidative Folding of Two‐Chain BI‐VI

The synthesized H‐ and L‐chains (50 nmol, each) were dissolved in a 25 mM sodium bicarbonate buffer at pH of 10.0 (50 µL) containing 1 mM EDTA. Using the same buffer, 25 mM GSH and 5 mM GSSG solutions were also prepared. On an ice bath, the H‐chain solution (50 µL) was mixed with the L‐chain solution (50 µL), the GSH solution (20 µL), the GSSG solution (20 µL), and the buffer solution (110 µL). The concentrations of H‐chain, L‐chain, GSH, and GSSG in the folding solution (250 µL) were 200 µM, 200 µM, 2 mM, and 0.4 mM, respectively. The mixture was then incubated at 4 °C.

To monitor the reaction progress, small aliquots (5 µL) were taken from the mixture, quenched by addition of aqueous AEMTS solution (7 mg/mL, 200 µL), diluted with 0.1% TFA (800 µL) in water, and analyzed by RP‐HPLC. After completion of the reaction, the folding mixture was added with an AEMTS solution (1 mL) to quench the folding. Generated BI‐VI was isolated by HPLC and lyophilized to obtain as white powder. The product was characterized by ESI‐TOF‐MS and amino acid analysis (AAA). The percentage of yields of BI‐VI under several NCA conditions were summarized in Table 1. ESI‐TOF mass, found: *m*/*z* 1174.90, calcd: 1174.50 for [M+5H]^5+^. Amino acid analysis: Asp_5.06_ Thr_3.68_ Ser_2.62_ Glu_4.33_ Pro_2.68_ Gly_2.14_ Ala_2_ Val_1.84_ Ile_1.69_ Leu_2.95_ Tyr_3.75_ Phe_2.00_ Lys_4.85_ Arg_0.98_.

### HPLC Analysis

The HPLC system was equipped with a sample solution loop (1 or 5 mL) and a TSKgel ODS‐100 V φ 4.6 ×150 mm RP column (Tosoh Corporation, Japan), which were incubated in a column oven at 35 °C with a flow rate of 0.5 mL mi^−1^n. The peptide chains as well as the folding products were detected by UV absorption at 220 nm. Solvent gradients were applied using eluent A (0.1% TFA in water) and eluent B (0.1% TFA in CH_3_CN). The gradient conditions are shown in the caption of Figure 3 or directly on the HPLC charts of Supporting Information.

### CD Spectral Measurement

Far‐UV circular dichroism (CD) spectra were measured with a J‐1500 spectrophotometer (JASCO, Japan) using a quartz cuvette with a 0.1 cm path length. According to the literature,^[^ ^41^ ^]^ the isolated native BI‐VI (14.5 nmol) was dissolved in a 10 mM sodium acetate buffer at pH of 3.9 containing 0.1 M NaCl (352 µL). The CD spectrum of the BI‐VI solution (42.1 µM) was measured at 37 °C between the wavelengths of 200 and 260 nm, applying 1 nm bandwidth, 1 s response, 20 nm/min scan rate, and 16 scan times. The CD spectral changes during the folding of BI‐VI were recorded as follows. An aliquot (20 µL) was taken from the folding solution and added to a 10 mM sodium acetate buffer at pH of 3.9 containing 0.1 M NaCl (280 µL). The CD spectra were measured under the same conditions as above. The CD signals were expressed as the mean residue ellipticity, [*θ*] (degrees cm^2^ dmol^−1^).

### Inhibitory Assay

According to the literature method,^[^ ^32^ ^]^ the inhibitory activities of folded BI‐VI, as well as H‐ and L‐chains in the reduced form, against bromelain were measured. A McIlvaine buffer solution at pH of 4.6 was prepared by mixing NaH_2_PO_4_ and citric acid in water. Using this buffer solution, 1 mM BI‐VI, H‐chain, and L‐chain solutions were prepared. A 10 mM Boc‐Leu‐Arg‐Arg‐AMC (MCA) solution was prepared by dissolving MCA (1.1 mg) in DMSO (157 µL). To the McIlvaine buffer solution (20 mL) were added 2‐mercaptoethanol (1.4 µL) and 80 µM bromelain stock solution (10 µL), which was freshly prepared in the McIlvaine buffer, to prepare a 40 nM bromelain and 1 mM 2‐mercaptoethanol solution at pH of 4.6. In a quartz cuvette (4 sides transparent with 1×1 cm path lengths), the prepared solution (1 mL) was diluted with the McIlvaine buffer solution at pH 4.6 (1 mL) and then added with the sample solution (0 µL for a blank, 10 µL for BI‐VI and H‐ and L‐chains). The hydrolysis reaction was started by adding the MCA solution (10 µL) to the mixture. The concentrations of a test sample (BI‐VI, A‐chain, or B‐chain), bromelain, MCA, and 2‐mercaptoethanol in the reaction solution were 5 µM, 20 nM, 50 µM, and 0.5 mM, respectively. Since the enzymatic activity of bromelain decreased gradually at room temperature probably due to air oxidation,^[^ ^34^ ^]^ bromelain was stored in a refrigerator after weighting and the time between the preparation of the bromelain stock solution and the start of the hydrolysis reaction was kept constant (15 minutes). After the incubation at room temperature for 10 minutes, the fluorescence spectrum (from 400 to 550 nm) was recorded on FP‐8600 Fluorescence Spectrometer (JASCO, Japan) applying an excitation wavelength of 370 nm. Inhibitory activities of the test samples against bromelain were estimated at 460 nm. The measurements were repeated three times.

### Statistical Analysis

The one‐way analysis of variance (ANOVA) was used to determine whether there are any statistically significant differences among the means of independent groups. Tukey's test was performed as a post‐hoc analysis to address the pairs of groups that are with significant difference. When *p* < 0.01, the differences were regarded as statistically significant. All statistical analyses were carried out by Microsoft Excel program using built‐in functions for one‐way ANOVA. For the post‐hoc test, the analysis was manually done using the same program.

## Supporting Information

Synthesis of H‐ and L‐chains of BI‐VI by SPPS (Figures S1–S3); Structural assignments for the folding intermediates of BI‐VI (Figure S4); Two‐chain folding of BI‐VI via NCA (Figures S5 and S6 and Table S1); Fluorescent spectra observed by the inhibitory assay for BI‐VI (Figure S7); ^1^H NMR spectra for H‐(Dmb)Gly‐OH and Fmoc‐Phe‐(Dmb)Gly‐OH (Figures S8 and S9).

## Conflict of Interests

The authors declare no conflict of interest.

## Supporting information



Supporting Information

## Data Availability

The data that support the findings of this study are available in the supporting information of this article.
