# Supplementary material for: Oxidative Folding of a Two‐Chain Protein Having Three Interchain Disulfide Bonds. Synthesis of Bromelain Inhibitor VI Through Native Chain Assembly
Source: Chemistry. 2025 May 20;31(37):e202500486. doi: 10.1002/chem.202500486 (PMC12223344; doi:10.1002/chem.202500486)
Supplement: Supplementary file 1 — Supporting Information [file CHEM-31-e202500486-s001.docx]

**Supporting Information**

B

A

C

**Figure S1.** Synthesis of the H-chain of BI-VI by simple SPPS. A: The HPLC chart after the cleavage from the resin. B: The MALDI-TOF-MS spectrum for peak A. C: The MALDI-TOF-MS spectrum for peak B. HPLC analysis conditions: column, a TSKgel ODS-100V φ 4.6 × 150mm RP column (Tosoh Corporation, Japan); detection, at 220 nm; solvent gradient, CH_3_CN 10 to 40 % in 25 min in the presence 0.1 % TFA; flow rate, 1.0 mL/min.

C

A

B

**Figure S2.** Synthesis of the H-chain of BI-VI by SPPS applying -Phe(Dmb)Gly-. A: The HPLC chart after the cleavage from the resin. A use of the TFA cocktail containing EDT resulted in an increase of the yield. B: The HPLC chart after purification. C: The ESI-TOF-MS spectrum with the expansions for the [M+4H]^4+^ and [M+3H]^3+^ peaks. HPLC analysis conditions: column, a TSKgel ODS-100V φ 4.6 × 150mm RP column (Tosoh Corporation, Japan); detection, at 220 nm; solvent gradient, CH_3_CN 10 to 40 % in 25 min in the presence 0.1 % TFA; flow rate, 1.0 mL/min.

A

B

C

**Figure S3.** Synthesis of the L-chain of BI-VI by SPPS. A: The HPLC chart after the cleavage from the resin. B: The HPLC chart after purification. C: The MALDI-TOF-MS spectrum. HPLC analysis conditions: column, a TSKgel ODS-100V φ 4.6 × 150mm RP column (Tosoh Corporation, Japan); detection, at 220 nm; solvent gradient, CH_3_CN 10 to 40 % in 25 min in the presence 0.1 % TFA; flow rate, 1.0 mL/min.

D

A

E

C

B

**Figure S4.** Structural assignments for the folding intermediates of BI-VI observed during NCA. A: The HPLC chart for the folding mixture after 1 d. The NCA conditions were the same the Figure 3. B: The MALDI-TOF-MS spectrum for R^L^. C: The MALDI-TOF-MS spectrum for 1SS^L^. D: The MALDI-TOF-MS spectrum for 2SS^H^. E: The MALDI-TOF-MS spectrum for 3SS^H^. HPLC analysis conditions: column, a TSKgel ODS-100V φ 4.6 × 150mm RP column (Tosoh Corporation, Japan); detection, at 220 nm; solvent gradient, CH_3_CN 10 to 40 % in 25 min in the presence 0.1 % TFA; flow rate, 1.0 mL/min.

C

A

B

**Figure S5.** Oxidative folding of BI-VI via NCA. A: The HPLC chart for the folding mixture after 10 d. The NCA conditions were the same the Figure 3. B: The HPLC chart after purification. HPLC analysis conditions: column, a TSKgel ODS-100V φ 4.6 × 150mm RP column (Tosoh Corporation, Japan); detection, at 220 nm; solvent gradient, CH_3_CN 10 to 40 % in 25 min in the presence 0.1 % TFA; flow rate, 1.0 mL/min. C: The ESI-TOF-MS spectrum of isolated BI-VI with the expansions for the [M+6H]^6+^, [M+5H]^5+^, [M+4H]^4+^, and [M+3H]^3+^ peaks.

**Table S1.** Supplementary summary of oxidative folding of BI-VI via native chain assembly (NCA). ^[a]^

| Entry | H-chain (μM) | L-chain (μM) | pH | Temp (°C) | Additives | Reaction time | Isolated yield (%) | HPLC yield (%) |
| --- | --- | --- | --- | --- | --- | --- | --- | --- |
| 1 [b] | 200 | 200 | 10.0 | ‒10 | 0.4 M urea, 4 mM GSH, 0.8 mM GSSG, 5 mM DTT, 10% EG | 7 w | 28 | 47 |
| 2 [c] | 200 | 200 | 10.0 | ‒10 | 2 mM GSH, 0.4 mM GSSG, 10% EG | 4 w | 32 | 58 |
| 3 [d] | 200 | 200 | 10.0 | 4 | 2 mM GSH, 0.4 mM GSSG | 2 w | 53 | 83 |
| 4 | 200 | 200 | 10.0 | 4 | 2 mM GSH, 0.4 mM GSSG | 2 w | – | 73 |
| 5 [e] | 200 | 200 | 10.0 | 4 | 2 mM GSH, 0.4 mM GSSG | 10 d (CD) | – | 80 |
| 6 | 200 | 200 | 10.0 | 4 | 2 mM GSH, 0.4 mM GSSG | 8 w | 43 | 68 |
| 7 | 200 | 200 | 10.0 | 4 | 2 mM GSH, 0.4 mM GSSG, 1.0 mM DTT | 4 w | – | 65 |
| 8 | 200 | 200 | 10.0 | 4 | 2 mM GSH, 0.4 mM GSSG, 1.0 mM DTT | 5 w | 31 | 47 |
| 9 [f] | 200 | 200 | 10.0 | 4 | 1.0 mM DTT | 4 w | 36 | 72 |

[a] GSH, glutathiones; GSSG, glutathione disulfide; DTT, dithiothreitol; EG, ethylene glycol.

[b] = Table 1, entry 1. [c] = Table 1, entry 2. [d] = Table 1, entry 3. [e] = Figure 3. [f] = Table 1, entry 4.

A

C

B

**Figure S6.** Series of HPLC charts obtained with different folding times during oxidative folding of BI-VI via NCA. A: Under the conditions of Table S1, entry 3 (= Table 1, entry 3). B: Under the conditions of Table S1, entry 7 (=Table S1, entry 3). C: Under the conditions of Table S1, entry 9 (= Table 1, entry 4). Structure for each peak was assigned by MALDI-TOF-MS analysis. HPLC analysis conditions: column, a TSKgel ODS-100V φ 4.6 × 150mm RP column (Tosoh Corporation, Japan); detection, at 220 nm; solvent gradient, CH_3_CN 10 to 40 % in 25 min in the presence 0.1 % TFA; flow rate, 1.0 mL/min.

Relative Fluorescence Intensity

Relative Fluorescence Intensity

Relative Fluorescence Intensity

**Figure S7.** Results of inhibitory assay for the synthesized BI-VI, reduced H-chain, and reduced L-chain against bromelain using Boc-Leu-Arg-Arg-AMC (MCA) as a substrate. The relative activities were determined by the fluorescent intensity at 440 nm. See the experimental section for details of the assay conditions.

***f***

***e***

***e***

***f***

***g***

***d***

***g***

***d***

***c***

***c***

***b***

***b***

***a***

***a***

**Figure S8.** 500 MHz ^1^H NMR spectrum for H-(Dmb)Gly-OH in CDCl_3_.

B

***(d+g+l+m)***

B

B

***i***

***(e+f)***

C

C

A

***h***

***b+c***

***b+c***

***h***

***(a+k+n)***

A

***j***

***j***

x

**Figure S9.** 500 MHz ^1^H NMR spectrum for Fmoc-Phe-(Dmb)Gly-OH in CDCl_3_.
